# Bacterial Synergism in Lignocellulose Biomass Degradation – Complementary Roles of Degraders As Influenced by Complexity of the Carbon Source

**DOI:** 10.3389/fmicb.2017.01628

**Published:** 2017-10-10

**Authors:** Larisa Cortes-Tolalpa, Joana F. Salles, Jan Dirk van Elsas

**Affiliations:** Department of Microbial Ecology, Groningen Institute for Evolutionary Life Sciences, University of Groningen, Groningen, Netherlands

**Keywords:** lignocellulose degradation, microbial consortia, synergism, wheat straw, recalcitrance, carbon sources

## Abstract

Lignocellulosic biomass (LCB) is an attractive source of carbon for the production of sugars and other chemicals. Due to its inherent complexity and heterogeneity, efficient biodegradation requires the actions of different types of hydrolytic enzymes. In nature, complex microbial communities that work efficiently and often synergistically accomplish degradation. Studying such synergisms in LCB degradation is fundamental for the establishment of an optimal biological degradation process. Here, we examine the wheat straw degradation potential of synthetic microbial consortia composed of bacteria and fungi. Growth of, and enzyme secretion by, monocultures of degrader strains were studied in aerobic cultures using wheat straw as the sole carbon and energy source. To investigate synergism, co-cultures were constructed from selected strains and their performance was tested in comparison with the respective monocultures. In monoculture, each organism – with a typical enzymatic profile – was found to mainly consume the cellulose part of the substrate. One strain, *Flavobacterium ginsengisoli* so9, displayed an extremely high degradation capacity, as measured by its secreted enzymes. Among 13 different co-cultures, five presented synergisms. These included four bacterial bicultures and one bacterial–fungal triculture. The highest level of synergism was found in a *Citrobacter freundii*/*Sphingobacterium multivorum* biculture, which revealed an 18.2-fold increase of the produced biomass. As compared to both monocultures, this bacterial pair showed significantly increased enzymatic activities, in particular of cellobiohydrolases, mannosidases, and xylosidases. Moreover, the synergism was unique to growth on wheat straw, as it was completely absent in glucose-grown bicultures. Spent supernatants of either of the two partners were found to stimulate the growth on wheat straw of the counterpart organism, in a directional manner. Thus, the basis of the LCB-specific synergism might lie in the specific release of compounds or agents by *S. multivorum* w15 that promote the activity of *C. freundii* so4 and vice versa.

## Introduction

Millions of tons of agricultural waste are generated globally every year (Väisänen et al., 2016). Examples are wheat and maize straws, sugarcane bagasse and corn stover. Such lignocellulosic biomass (LCB) is useful as raw material for the production of value-added materials as well as fuels. LCB is composed of lignin, cellulose and hemicellulose, whereas pectin, proteins, small molecules, and minerals can also be present (Guerriero et al., 2016). The exact composition of LCB depends on factors such as plant cultivar type, plant age, local growth conditions, harvesting season and the quality of the soil used for cultivation. For instance, depending on cultivar, age and local conditions, wheat straw can contain 30–44% cellulose, 23–50% hemicellulose, and 7.7–15% lignin (Van Dyk and Pletschke, 2012). A clear impediment to the widespread use of wheat straw as raw material for value-added compounds is its relatively recalcitrant nature, which means it does not easily break down into its monomers. This recalcitrance is clearly caused by its complex chemical composition, and it relates to a major extent to the tight linkages between the lignin, hemicellulose, and cellulose parts. Moreover, the LCB physical structure, i.e., the degree of crystallinity and polymerization of cellulose and polysaccharide, is an important parameter that influences its degradability (Van Dyk and Pletschke, 2012; Bhattacharya et al., 2015).

As a reflectance of its inherent complexity, a large variety of organisms (producing diverse enzymes) is commonly needed to efficiently degrade LCB like into its monomer compounds. In nature, microbial communities commonly degrade it in a dynamic and time-dependent manner. The degraders are thus presumed to show dynamic responses to the substrate, reaching higher biomass when working together when than acting alone. This process is known as synergistic growth. Moreover, the degrading organisms may use enzymes with complementary activities (enzymatic synergism). Synergism in growth and that in enzymatic activity therefore reflect two processes that are often closely linked in microbial communities (Van Dyk and Pletschke, 2012; Cragg et al., 2015). We took these two definitions into our own work on microbial consortia, as proposed in the recent literature (Mitri and Foster, 2013; Deng and Wang, 2016). Given the fact that in natural systems synergism in LCB degradation processes is the rule rather than the exception, we surmised it is exacerbated in soil-derived microbial consortia selected on LCB.

What mechanisms are behind synergistic behavior in LCB degradation? According to classical knowledge and theory, microorganisms growing together on one substrate, when coexisting, most often divide labor, in a process called niche partitioning. Metabolic complementarity is the main process behind such niche partitioning, as revealed by the classical example of biofuel and hydrogen production through co-cultures of *Bacillus* and *Clostridium* on rice straw compost (Chang et al., 2008). So far, it has been relatively unknown to what extent complex substrates like LCB foster processes leading to coexistence. However, recently a co-culture of *Trichoderma reesei* and *Escherichia coli* growing on (pretreated) corn stover was found to be optimal in isobutanol production (Minty et al., 2013). The strategy was based on division of function between the two organisms. *T. reesei* secreted cellulolytic enzymes that transformed the LCB into soluble saccharides, whereas *E. coli* fermented these into isobutanol. Another recent study reported that, along the same lines, co-cultures of *Clostridium cellulovorans* (743B) and *C. beijerinckii* (NCIMB 8152) also successfully produced butanol, under mesophilic conditions (Wang et al., 2015). These studies thus show the key importance of metabolic complementarity in LCB degradation, in which the cooperation between synergistic pairs is driven by exchanges of key metabolites, or by niche partitioning. However, we still do not understand the plethora of mechanisms, as well as the dynamism, that play roles in the microbial attack on the LCB wheat straw (Pandhal and Noirel, 2014; Dolinšek et al., 2016; Ghosh et al., 2016; Jia et al., 2016; Jiang et al., 2017). For instance, it remains unclear to what extent the composition/structure of the substrate affects the interactions between collaborating degraders. Moreover, the dynamism in the interactions and activities of collaborative organisms remains understudied.

In our previous work, a suite of microbial strains was isolated from three lignocellulolytic microbial consortia that had been selected by repeated growth on raw wheat straw as the single carbon and energy source. Most of the strains had shown promising lignocellulolytic capabilities (Cortes-Tolalpa et al., 2016). We here hypothesized that the wheat straw substrate, being complex and spatially structured, will promote ‘division of labor,’ and so cooperation, between some of the degrader strains. The aim of this study was, therefore, to uncover such synergisms and determine their potential. In this endeavor, we also addressed the potential mechanism behind the synergisms. The data showed that cooperative behavior was relatively ‘common’ in microbial consortia growing on wheat straw, but broke down when strain combinations were grown on simple substrates like glucose.

## Materials and Methods

### Bacterial and Fungal Strains

The bacterial and fungal strains used in this study were isolated from three wheat-straw-grown microbial consortia that had originally been inoculated with forest soil, canal sediment and decaying wood derived microbiomes. Briefly, serial dilutions of extracts of the aforementioned biomes were prepared in saline (0.85%). Then, 100 μL aliquots of each dilution were spread onto the surface of R2A (BD Difco, Detroit, MI, United States) and potato dextrose agar (PDA) plates, to isolate fungi and bacteria, respectively. Morphological differences of the colonies were used in the selection procedure of the isolates, which were streaked to purity and then preserved at -80°C (in LB broth with 20% glycerol and potato dextrose broth for bacteria and fungi, respectively). *Coniochaeta ligniaria* sedF1 reflected a dominant colony in the PDA plates, and so was thought to represent the main viable fungus (Cortes-Tolalpa et al., 2016).

### Culture Media

Three media, based on mineral medium (below) were used, on the basis of three different carbon sources. These were (1) “raw wheat straw” (1% w/v), (2) “synthetic recalcitrant biomass” (SRB) [0.3% carboxymethyl cellulose (CMC) (VWR, Leuven, Belgium), 0.5% xylan-beechwood (Sigma–Aldrich, Darmstadt, Germany) and 0.1% lignin (Sigma–Aldrich, St. Louis, MO, United States)] and (3) “glucose” (0.3%) (Merck, Darmstadt, Germany). The raw wheat straw was air-dried (50°C) before cutting it into pieces of about 5 cm length. Then, the pieces were thoroughly ground, using a mill hammer, to pieces ≤ 1 mm. No pre-treatment was performed (untreated raw substrate). All carbon sources were taken up in mineral medium [7 g/L Na_2_HPO_4_⋅2H_2_O; 2 g/L K_2_HPO_4_; 1 g/L (NH_4_)_2_SO_4_; 0.1 g/L Ca (NO_3_)_2_⋅4H_2_O; 0.2 g/L MgCl_2_⋅6H_2_O g/L, pH 7.2] (Jiménez et al., 2013; de Lima Brossi et al., 2015; Cortes-Tolalpa et al., 2016) supplemented with vitamin solution (0.1 g Ca-pantothenate, 0.1 g cyanocobalamine, 0.1 g nicotinic acid, 0.1 g pyridoxal, 0.1 g riboflavin, 0.1 g thiamin, 0.01 g biotin, 0.1 g folic acid; H_2_O 1 L) and trace metal solution (2.5 g/L EDTA; 1.5 g/L FeSO_4_⋅7H_2_O; 0.025 g/L CoCl_2_; 0.025 g/L ZnSO_4_⋅7H_2_O;0.015 g/L MnCl_2_; 0.015 g/L NaMoO_4_⋅2H_2_O; 0.01 g/L NiCl_2_; 0.02 g/L H_3_BO_3_; 0.005 g/L CuCl_2_). Sterility of the substrate was verified following plating on trypticase soy agar (TSA) plates. All chemicals and reagents used in this work were of analytical molecular biology grade (Sigma–Aldrich, Darmstadt, Germany). Erlenmeyer flasks containing 25 mL of the media were autoclaved at 121°C for 27 min before use.

### Monocultures and Co-cultures

Monoculture refers to the microbial strains growing alone in a flask. Co-culture refers to combined strains growing in a flask. Triplicates were used throughout. The selection of strains for the construction of the synthetic pairs was based on relative abundance, enzymatic activity and antagonism assay data, as reported earlier (Cortes-Tolalpa et al., 2016). After a first screening (**Table 1**), six bacterial and one fungal strain(s) were selected to examine the behavior in co-cultures. Thus 13 co-cultures were formed (**Table 2**).

**Table 1:** Taxonomic affiliation of microbial strains used in this study.

Taxonomy affiliation					

**Strain**	**Closest relative**	**Class**	**Family**	**^∗^Identity (%)**	**Accession number**
w4	*Chryseobacterium taihuense*	*Flavobacteria*	*Flavobacteriaceae*	99	KT265756
so3	*Chryseobacterium taihuense*	*Flavobacteria*	*Flavobacteriaceae*	98	KT265758
so4	*Citrobacter freundii*	*Gammaproteobacteria*	*Enterobacteriaceae*	99	KT265771
so22	*Sphingobacterium multivorum*	*Sphingobacteria*	*Sphingobacteriaceae*	98	KT265750
w15	*Sphingobacterium multivorum*	*Sphingobacteria*	*Sphingobacteriaceae*	97	KT265748
se10	*Sphingobacterium faecium*	*Sphingobacteria*	*Sphingobacteriaceae*	98	KT265798
w6	*Flavobacterium ginsengisoli*	*Flavobacteria*	*Flavobacteriaceae*	99	KT265792
so9	*Flavobacterium ginsengisoli*	*Flavobacteria*	*Flavobacteriaceae*	99	KT265787
so11	*Flavobacterium banpakuense*	*Flavobacteria*	*Flavobacteriaceae*	99	KT265796
so1	*Acinetobacter johnsonii*	*Gammaproteobacteria*	*Moraxellaceae*	99	KT265766
se1	*Acinetobacter beijerinckii*	*Gammaproteobacteria*	*Moraxellaceae*	99	KT265764
so5	*Comamonas testosteroni*	*Betaproteobacteria*	*Comamonadaceae*	99	KT265795
so16	*Ochrobactrum thiophenivorans*	*Alphaproteobacteria*	*Brucellaceae*	99	KT265790
se5	*Oerskovia enterophila*	*Actinobacteria*	*Cellulomonadaceae*	99	KT265785
so12	*Lelliottia amnigena*	*Gammaproteobacteria*	*Enterobacteriaceae*	99	KT265765
so14	*Microbacterium oxydans*	*Actinobacteria*	*Microbacteriaceae*	99	KT265770
so24	*Stenotrophomonas rhizophila*	*Gammaproteobacteria*	*Xanthomonadaceae*	99	KT265769
w1	*Achromobacter xylosoxidans*	*Betaproteobacteria*	*Alcaligenaceae*	99	KT265794
w5	*Delftia tsuruhatensis*	*Betaproteobacteria*	*Comamonadaceae*	99	KT265782
w8	*Microbacterium gubbeenense*	*Actinobacteria*	*Microbacteriaceae*	97	KT265752
w9	*Microbacterium foliorum*	*Actinobacteria*	*Microbacteriaceae*	99	KT265781
w13	*Raoultella terrigena*	*Proteobacteria*	*Enterobacteriaceae*	99	KT265761
w16	*Stenotrophomonas terrae*	*Gammaproteobacteria*	*Xanthomonadaceae*	99	KT265788
^∗∗^sedF1	*Coniochaeta ligniaria*	*Sordariomycetes*	*Coniochaetaceae*	96	KT265807

^∗^*Closest relative species. According to 16S or 18S (^∗∗^) ribosomal RNA gene sequence comparisons*.

**Table 2:** Microbial composition of the co-cultures in this study.

Taxonomy affiliation				

**Co-culture**	**Strain code**	**Strain 1**	**Strain 2**	**Strain 3**
A	so4, w15	*C. freundii* so4	*S. multivorum* w15	
B	so4, so22	*C. freundii* so4	*S. multivorum* so22	
C	so4, so1	*C. freundii* so4	*A. johnsonii* so1	
D	w15, so1	*S. multivorum* w15	*A. johnsonii* so1	
E	so9, so1	*F. ginsengisoli* so9	*A. johnsonii* so1	
F	so4, so9	*C. freundii* so4	*F. ginsengisoli* so9	
G	w15, so9	*S. multivorum* w15	*F. ginsengisoli* so9	
H	so4, w9	*C. freundii* so4	*M. foliorum* w9	
I	w15, w9	*S. multivorum* w15	*M. foliorum* w9	
J	so4, w15, so1	*C. freundii* so4	*S. multivorum* w15	*A. johnsonii* so1
K	so4, w15, so9	*C. freundii* so4	*S. multivorum* w15	*F. ginsengisoli* so9
L	so4, w15, sedF1	*C. freundii* so4	*S. multivorum* w15	*C. ligniaria* sedF1
M	so4, w15, w9	*C. freundii* so4	*S. multivorum* w15	*M. foliorum* w9

### Microbial Culture and Growth Measurements

The mono- and co-cultures were grown in Erlenmeyer flasks (in triplicates). To prepare inocula, microbial strains were pre-grown on TSA plates at 28°C for 48 h. Then a fresh colony of each strain was dissolved in sterile saline (0.85% NaCl). The fungal strain was first adapted to growth in liquid media (potato dextrose broth) for 48 h. The optical density of the bacterial and fungal suspensions were then checked, after which they were adjusted to that representing a standard cell density of about 5 log cells per mL. The incubation conditions were 28°C with shaking at 180 rpm. Microbial growth was measured at regular time points, i.e., every 24 h until 72 h. At each time point, 1 mL culture was harvested, cells were spun down (20 min, 13,300 rpm, 4°C – Eppendorf centrifuge, Hamburg, Germany), and the supernatant was used for enzymatic activity analyses. Then, cells were resuspended in sterile saline and the resulting suspensions used for serial dilution plating on TSA. The inoculated plates were incubated at 28°C for 24–48 h, after which the developed colonies were counted. Thus, growth was monitored by CFU counting following incubation. To determine the maximal growth rates of the cultures (μ, h^-1^), the numbers of CFUs measured during the exponential growth phase were log-transformed and the slope of each growth curve was used. Flasks with culture medium without cells were used as negative controls (NCs).

### Lignocellulolytic Enzyme Activity Assays

The activities of four different enzymes were monitored at time points 0, 24, 48, and 72 h. Substrates for β-glucosidase (BG) (EC. 3.2.1.37), cellobiohydrolase (CBH) (EC. 3.2.1.91), β-mannosidase (BM) (3.2.1.25), and β-xylosidase (BX) (EC. 3.2.1.37) activities were used. The first two substrates report on the degradation of cellulose and the last two on that of the hemicellulose part of wheat straw. The activities were quantified on the basis of the (enzyme-specific) substrate label 4-methylumbelliferone (MUB): 4-MUB-β-glucosidase, 4-MUB-β-cellobiosidase, 4-MUB-β-mannosidase, and 4-MUB-β-xylosidase (Sigma–Aldrich, Darmstadt, Germany). The reaction mixtures consisted of 150 μL diluted supernatant (usually 1/4) in MOPS buffer (50 mM, pH 6.5; Sigma–Aldrich, Darmstadt, Germany) and 2 mM of MUB substrate in black 96-well plates. The reactions were incubated 1 h at 28°C in the dark, after which 30 μL of NaOH (1 M) was added. Fluorescence was measured at an excitation wave length of 365 nm with emission at 445 nm. The enzymatic activities were then calculated from the fluorescence units using a standard calibration curve. Supernatant recovered from the NC was also tested, and thus served as the NC. The enzymatic activities are reported as the rate of MUB production (nmol MUB per h at 28°C, pH 6.8). All assays were done in triplicate.

### Antagonistic Interaction Assays

Antagonistic interactions were tested with Burkholder’s ‘spot-on-lawn’ method (Burkholder et al., 1966). Strains were confronted with each other in a set-up to obtain a full interaction matrix of all strains with each other. Lawns of each strain were created by mixing exponentially grown cultures (optical density 0.5 at 600 nm) with soft carboxymethyl cellulose (CMC)-xylan agar media (CMC 0.2%, xylan 0.1%, yeast extract 0.05%, 1.5% agar) and pouring these onto the surface of LB agar plates. Following solidification, five microliters of overnight cultures of selected bacterial or fungal strains were added on top (Pérez-Gutiérrez et al., 2013; Aguirre-von-Wobeser et al., 2014). The plates were incubated for 48 h at 28°C, after which they were inspected for inhibition haloes around the growth of the test strains. The broad-spectrum antibiotic streptomycin was used as a control (data not shown).

### Synergism

The degree of enzymatic synergism (DS) (Van Dyk et al., 2013) was calculated by dividing the observed enzymatic activity from each co-culture (secretome) by the sum of the individual activities of the secretome from the respective monocultures. Greater values of the calculated DS indicate a greater enzymatic synergism. Synergistic growth was defined as having occurred when the biomass developed in the co-culture was significantly (*t*-test, *P* < 0.05) higher than the sum of the biomasses achieved in the respective monocultures.

### Induction Experiment

Monocultures of strains *Sphingobacterium multivorum* w15 and *Citrobacter freundii* so4 were prepared as described above, using either raw wheat straw or glucose as the carbon source. Supernatants were harvested and filtered (0.2-μm pore size filter). No viable cells were detected in the supernatants. For the induction of strain w15, 10% of the final volume of *C. freundii* so4 culture supernatant was added to the *S. multivorum* w15 culture. Moreover, *C. freundii* so4 was treated in the reciprocal way. For both cultures, the supernatants were added at the onset of the incubation. Triplicate treatments were used. The controls consisted of strains growing with the addition of 10% of the medium. The growth and enzymatic activities were then monitored over time and compared with their respective controls.

### Statistical Analyses

For the detection of differences in growth across the cultures, we used Student’s *t*-test. Since the enzymatic activity data had a non-normal distribution, even after log transformation (x+1), we used the non-parametric Kruskal–Wallis test. Regression analyses between monoculture growth rates and enzymatic activities were performed in SPSS (data not shown). Data were considered to be significant at *P* < 0.05.

## Results

### Testing for Potential Antagonisms on CMC-Xylan Agar Media

The strains used in these tests are shown in **Table 1**. Testing for antagonism across all pairs of strains revealed that, under the conditions used, none of the bacterial strains exhibited antagonism to any of the other strains (data not shown). Considering the fungal strain *C. ligniaria* sedF1, we found no antagonistic effect of it on any of the bacterial strains.

### Monocultures

**FIGURE 1:**
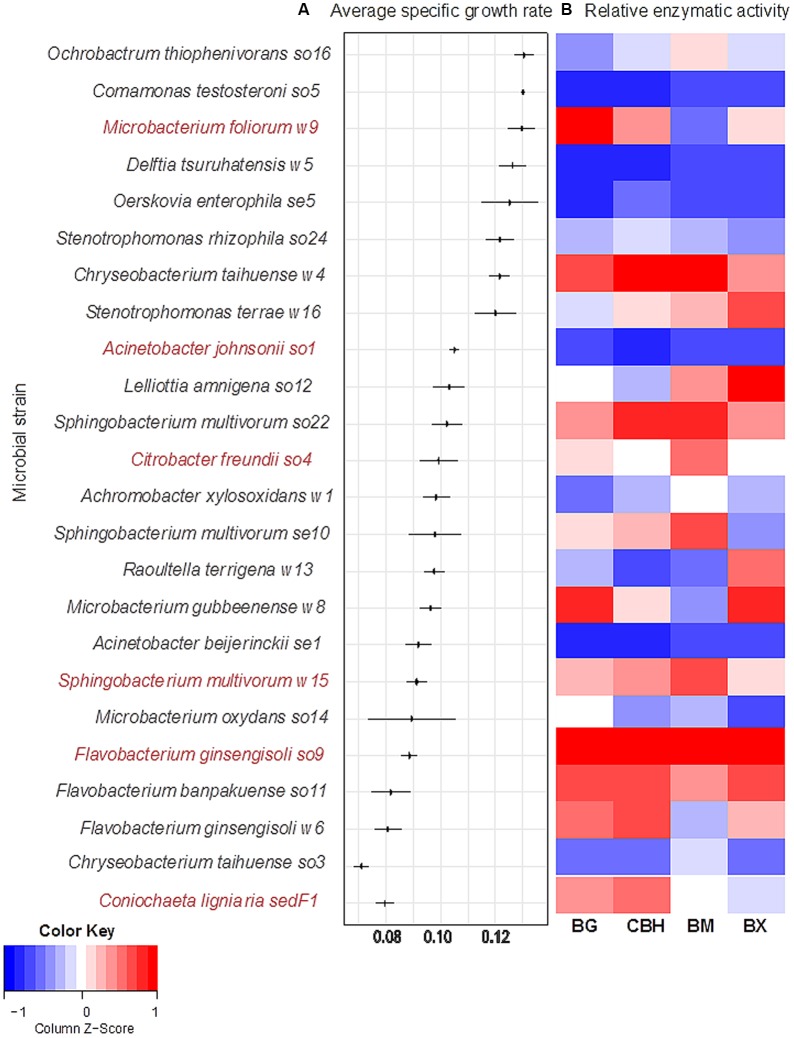
Screening of growth and degradation capacity of selected microbial strains. Microbial strains were isolated from three different enriched consortia. **(A)** The left panel shows specific growth rates, μ (h^-1^), in decreasing order. Horizontal line represents standard deviation across triplicates. Selected strains are shown in red. **(B)** The right panel shows relative activity of four lignocellulolytic enzymes, BG, β-glucosidase; CBH, cellobiohydrolase; BM, β-mannosidase; and BX, β-xylosidase. The relative enzymatic activity is reported in nmol MUB released per h at 28°C, pH 6.8. Activity values are normalized by using log (x+1).

Twenty-three among 51 bacterial strains obtained from the wheat straw microbial consortia (**Table 1**) were able to grow aerobically in monoculture in minimal medium with wheat straw as the sole source of carbon and energy. All of the 23 growth-positive bacterial cultures grew from a start density of around 5, to a final density of around 8 log cell/mL after 48–72 h. The strains revealed different specific growth rates, expressed as μ (h^-1^) (**Figure 1**). The fungal strain *C. ligniaria* sedF1 also grew well. We decided to work further with these bacterial strains, omitting the 28 non-growers from this study. In addition, we included the fungal strain *C. ligniaria* sedF1 on the basis of the prevalence of this fungal species across all wheat straw grown enrichments.

#### Growth Rates

The specific growth rates, expressed as μ (h^-1^), of the 23 bacterial strains (**Table 1**), next to that of the single fungal strain can be seen in **Figure 1**. Three main groups were observed, typified by either high, intermediate or low growth rates. Eight strains fell in the high-growth-rate class [average μ = 0.13 h^-1^ (±0.0013)]. These were: *Ochrobactrum thiophenivorans* so16, *Comamonas testosteroni* so5, *Microbacterium foliorum* w9, *Delftia tsuruhatensis* w5, *Oerskovia enterophila* se5, *Stenotrophomonas rhizophila* so24, *Chryseobacterium taihuense* w4 and *Stenotrophomonas terrae* w16. The second group, composed of 12 strains, revealed intermediate growth rates [i.e., μ = 0.10 h^-1^ (±0.005)]. These were *Acinetobacter johnsonii* so1, *Lelliottia amnigena* so12, *S. multivorum* so22, *C. freundii* so4, *Achromobacter xylosoxidans* w1, *S. multivorum* se10, *Raoultella terrigena* w13, *Microbacterium gubbeenense* w8, *Acinetobacter beijerinckii* se1, *S. multivorum* w15, *Microbacterium oxydans* so14, and *Flavobacterium ginsengisoli* so9. The remaining two bacterial strains (as well as the fungus) grew slowly, with a μ of 0.08 ± 0.006 h^-1^. These were *F. banpakuense* so11 and *C. taihuense* so3, next to *C. ligniaria* sedF1 (**Table 1** and **Figure 1A**).

#### Degradation Potential

We examined the production of extracellular β-glucosidases, cellobiohydrolases, β-mannosidases, and β-xylosidases in each of the monocultures. The data show that only five strains (*A. johnsonii* so1, *A. beijerinckii* se1, *C. testosteroni* so5, *O. enterophila* se5, and *D. tsuruhatensis* w5) did not yield any enzymatic activity on the four substrates (**Figure 1B**). For the remaining 18 bacterial and one fungal strain, specific suites of released enzymes were found (**Figure 1B**). For all enzymes, the total activities measured consistently increased over time, being maximal at 72 h (**Figure 1**). This indicated growth-related enzyme secretion across all these strains.

However, none of the monocultures showed a clear relationship between enzymatic activity and growth rate (using regression analysis) (data not shown). For instance, *C. testosteroni* so5, *O. enterophila* se5, and *D. tsuruhatensis* w5 revealed high growth rates on the wheat straw, but they did not reveal activity on any of the enzyme substrates (**Figure 1**). On the other hand, the intermediate-growth-rate *F. ginsengisoli* so9 showed very high β-glucosidase, cellobiohydrolase, and β-xylosidase activities. In contrast, *O. thiophenivorans* so16 revealed the highest μ of all strains (0.13 h^-1^, ±0.005), whereas it revealed only intermediate values of the four enzymatic activities (**Figure 1B**).

### Co-cultures

Starting from the premise that bacteria, next to fungi, make up the major part of the wheat-straw-selected microbial consortia (Cortes-Tolalpa et al., 2016), we used educated guesses to assemble co-cultures with presumed collaborative substrate degradation activity. The co-cultures thus included a selection of highly performing or high-abundance bacteria, next to a dominant fungus (**Table 2**).

#### Bacterial Strain Selection

Combinations of strains were formed on the basis of (1) the abundance values of the respective bacterial types in the three source microbial consortia (Cortes-Tolalpa et al., 2016), (2) the performance of strains in the current tests of growth and enzymatic activity on wheat straw. Thus, the enzyme-active *C. freundii* so4, *S. multivorum* strains w15 and so22, and *A. johnsonii* so1 were selected (OTUs dominant in wood/soil derived wheat straw bred consortia, and *S. multivorum* also in the sediment-derived consortia) (**Table 3**). In addition, *F. ginsengisoli* so9 was also chosen because it revealed the highest enzymatic activities of all screened strains. Finally, *M. foliorum* w9, presented in low abundance, was included in the work because it revealed a high growth rate and maximal glucosidase activities when grown on wheat straw at all time points (**Figure 1B**).

**Table 3:** Relative abundance and growth of most abundant bacterial strains in the final consortia derived from decaying wood, forest soil, and canal sediment.

Selected bacteria strain	Relative abundance (%) in consortia derived from:^∗^		
Affiliation/code	Wood	Soil	Sediment
*C. freundii* – so4	19.3 ± 5.1	19.7 ± 3.9	<2
*S. multivorum* – w15/so22	18 ± 11	23.4 ± 3.7	8.4 ± 1.8
*A. johnsonii* – so1	11.8 ± 7.6	<2	<2
*F. gingengisoli* – so9	5.6 ± 2.2	5.7 ± 1.2	<2
*M. foliorum* w9	<2	<2	<2

^∗^*Taken from Cortes-Tolalpa et al. (2016)*.

#### Fungal Strain Selection

The fungal strain *C. ligniaria* sedF1 (dominant in the sediment-derived wheat-straw-bred consortia) was selected (see Materials and Methods), as it revealed growth on lignocellulose, with considerable activity of β-glucosidases (1023.0 ± 9.4) and cellobiohydrolases (156.9 ± 0.4) in monoculture. Moreover, previous work had shown that this fungus may promote bacterial growth by removal of toxic compounds on torrified grass (Trifonova et al., 2009). This fungus has consistently been isolated from LCB grown microbial cultures, as reported in several recent studies (Jiménez et al., 2013; de Lima Brossi et al., 2015; Cortes-Tolalpa et al., 2016); it may itself have an important role in wheat straw degradation.

#### Growth in Co-cultures

##### Bacterial–bacterial bicultures

From the 13 co-cultures, four bicultures (A, C, D, and J) revealed synergistic growth, as evidenced by comparing the growth in the biculture to that in the monocultures of each of the strains. Bicultures H, I, K, and M did not show synergistic growth (*t*-test, *P* > 0.05) (Supplementary Figure S1A), whereas bicultures B, E, F, and G exhibited a partial positive interaction. In the latter, only one of the strains in the pair benefited from being in the co-culture (Supplementary Figure S1A). These were, for bicultures E, F, and G (in which the strong enzyme producer *F. ginsengisoli* so9 was involved): strains so1, so4, and w15, respectively. In the case of biculture B, both strains so4 and so22 revealed enhanced growth (as compared to the monoculture counterparts), although this was not significant (Supplementary Figure S1A).

**FIGURE 2:**
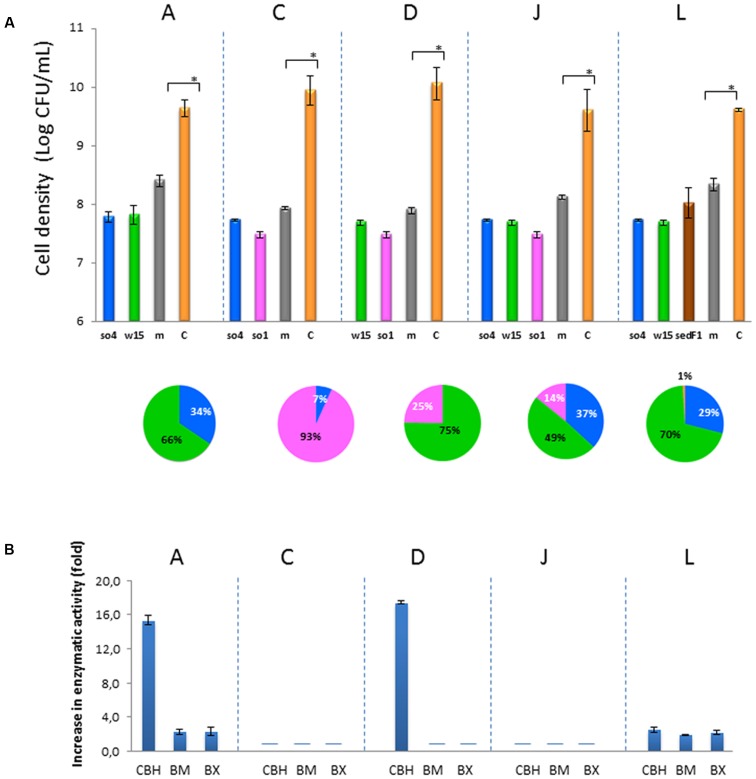
Characterization of synergistic co-cultures. **(A)** Cell densities (log CFU/mL) after 48 h. Significant differences between the sum of monocultures and co-cultures (*t*-test, *P* < 0.05) shown by ^∗^. Explanation: *m*, sum of monocultures (gray-m); and *C*, co-cultures (yellow-C). Explanation: so4, *C. freundii*, w15, *S. multivorum*, so1, *A. johnsonii*, sedF1, *C. ligniaria*. In the pie chart, the proportions of the individual strains in the co-culture at the end of the culture are shown. **(B)** Synergistic enzymatic activities in the supernatant from synergistic co-cultures. Y-axis shows the increase (fold) in the enzymatic rate in the co-culture compared with that in the separate monocultures (summed). X-axis shows the respective enzymatic assay. CBH, cellobiohydrolase; BM, β-mannosidase; BX, β-xylosidade. Only co-cultures A, D, and L presented synergistic enzymatic activities with the tested enzymes. Enzymatic activity data were based on nmol MUB produced per h at 28°C, pH 6.8. Bars indicate standard deviations across triplicate systems. (-) below detection.

The synergistic bicultures with highest gain in biomass were: A (*C. freundii* so4/*S. multivorum* w15), C (*C. freundii* so4/*A. johnsonii* so1), D (*S. multivorum* w15/*A. johnsonii* so1), and J (*C. freundii* so4/*S. multivorum* w15, *A. johnsonii* so1) (*t*-test, *P* < 0.05) (**Figure 2A**). Culture A revealed an increase of 18.2 (±0.3)-, C of 18.3 (±1.3)-, D of 20.5 (±0.6)-, and J of 15.3 (±2.4)-fold.

##### Bacterial-bacterial-fungal triculture

Only one bacterial-fungal triculture revealed synergistic growth (L). Triculture L, assembled by mixing *C. freundii* so4 S. *multivorum* w15 and *C. ligniaria* sedF1, revealed quite interesting results, as both bacterial strains exhibited synergistic growth in the presence of the fungus. In contrast, the fungus performed better in the monoculture (*t*-test, *P* < 0.05). Thus, in the triculture *C. freundii* so4 showed a growth increase of 27.8 (±0.8) and *S. multivorum* of 28.2 (±1.5) fold, compared to the respective monocultures. In contrast, the fungal strain showed a decrease in growth (43.9 ± 2.7 fold), compared with its biomass in monoculture.

#### Degradation Potential in Co-cultures

In most of the co-cultures, the production of β-glucosidases, cellobiohydrolases, β-mannosidases, and β-xylosidases was stimulated in a mixture- and time-dependent manner. This indicated mutual effects of the strains in spurring the production and/or secretion of lignocellulolytic enzymes. In other words, the activities measured in the co-cultures exceeded those found in the corresponding monocultures (**Figure 2B**).

Along the duration of the experiments, co-cultures C, H, J, K, L (**Table 2**) did not show any synergistic enzymatic activity. In contrast, cultures E and F (**Table 2**) displayed very high enzymatic activities at the end of the incubation period (72 h). Thus, measured activities were: 10351 ± 635.2 (for BG), 2205 ± 174.9 (for CBH), 5181.2 ± 847.9 (BG), and 515.4 ± 107.9 (for CBH), respectively (relative enzymatic activities reported in nmol of MUB released per h at 28°C, pH 6.8). The increased enzymatic activities were attributed to the presence of the high-enzyme producer *F. ginsengisoli* so9 across these cultures. Surprisingly, *F. ginsengisoli* so9 did not display any synergism in mixtures with other strains (Supplementary Figure S1B).

Among the five co-cultures that were synergistic for growth (**Table 2**), two did not show any synergistic enzymatic activities (C and J), whereas three others did (A, D, and L) (**Figure 2B**). In **Figure 2B**, we show the increase in enzymatic activities in the co-culture compared with the summed respective monocultures. For co-culture A, synergistic activities were found for cellobiohydrolases (DS_CBH_ = 15.3 ± 0.5), β-mannosidases (DS_BM_ = 2.3 ± 0.3), and β-xylosidases (DS_BX_ = 2.3 ± 0.5). Co-culture D exhibited exclusively (raised) cellobiohydrolase activities (DS_CBH_ = 17.4 ± 0.2). Concerning the two-bacterial-fungal co-culture L, synergism in the activities of cellobiohydrolases (DS_CBH_ = 2.0 ± 0.2), β-mannosidases (DS_BM_ = 1.9 ± 0.1), and β-xylosidases (DS_BX_ = 2.2 ± 0.2) were found (**Figure 2B**). Overall, the most ‘compatible’ biculture, in terms of enzymatic activities, was the system composed of *C. freundii* so4 and *S. multivorum* w15 (A). This system was ‘growth-synergistic,’ next to “enzyme-synergistic.” Interestingly, a clear commonality in co-cultures A, B, G, I, and M (which presented synergism in cellobiohydrolases, β-mannosidases, and β-xylosidases) was the presence of *S. multivorum* in the form of strains w15 or so22 (Supplementary Figure S1B).

### Influence of the Carbon Source on Collaboration between *C. freundii* so4 and *S. multivorum* w15

**FIGURE 3:**
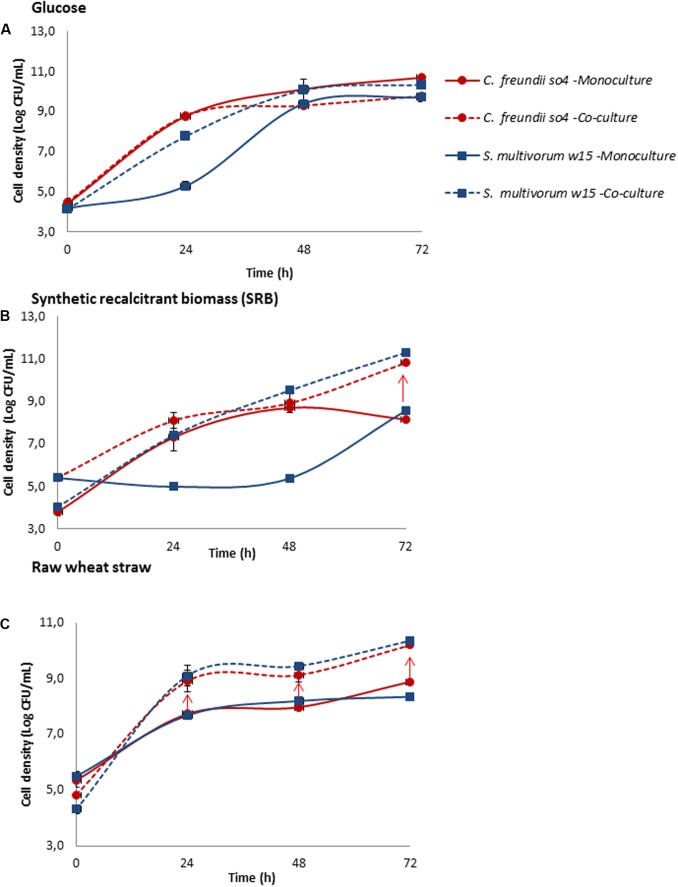
Influence of carbon source complexity on collaborative relationship between the most synergistic bacterial pair (*C. freundii* so4 / *S. multivorum* w15). *C. freundii* so4 (red) and *S. multivorum* w15 (blue) were grown in monoculture (-) and biculture (- -), on different carbon sources with different levels of “recalcitrance”: **(A)** glucose, **(B)** synthetic recalcitrant biomass (SRB, containing carboxymethyl cellulose [CMC], xylan-beechwood and lignin) and **(C)** raw wheat straw. *S. multivorum* w15, in monoculture, presented a long lag phase growing on glucose and SRB and immediate growth on raw wheat straw, whereas *C. freundii* so4 showed better adaptation to the synthetic medium. Both strains grew better in biculture. Y-axis: cellular density (log CFU/mL); X-axis: time in h. Red arrow indicates synergistic growth. Standard deviation based on triplicate systems - shown by vertical bars or within symbol dimensions.

To investigate if the carbon source has an influence on the collaborative behavior within bicultures, we selected the aforementioned most synergistic one, composed of *C. freundii* so4 and *S. multivorum* w15. Growth experiments were set up, in mono- and bicultures, on carbon sources with increasing levels of complexity and degradability, namely (1) glucose, (2) SRB (CMC, xylan, and lignin), and (3) wheat straw. Overall, the data revealed a strong relationship between the substrate type (see Materials and Methods) and the level of collaborative interaction in the system (**Figure 3**). Interestingly, in the biculture grown on glucose, no synergistic relationship was found (**Figure 3A**). When the strains were grown on SRB, synergistic growth was only observed at the end of the incubation period, i.e., after 72 h (**Figure 3B**). In sharp contrast, significant synergistic growth (*t*-test, *P* ≤ 0.05) along the incubation time was observed for the two strains growing together on the (raw) wheat straw (**Figure 3C**).

#### Specificity of Collaborative/Synergistic Growth

In the bicultures growing in SRB, after 72 h, C. freundii so4 showed an increase in density of 24.6 fold (±1.4), while *S. multivorum* w15 showed an increment of 24.2 fold (±7.9). Notably, the monoculture of the latter strain revealed a long lag phase, while *C. freundii* so4 did not reveal such a phenomenon (**Figure 3B**). Growing in biculture on raw wheat straw, after 24 h, *C. freundii* so4 presented an increase in density of 15.4 fold (±3.2), while *S. multivorum* w15 showed an increment of 19.4 fold ± 0.6 (**Figure 3C**). In contrast, there was no substantial fold increase in the bicultures grown on glucose for any of the two strains (**Figure 3A**). Hence, we posit that the level of recalcitrance of the substrate was congruent with the strength of the collaborative relationship between the two bacterial degraders.

#### Degradation Potential

*Citrobacter freundii* so4 and *S. multivorum* w15 growing in biculture in SRB did not exhibit synergistic enzymatic activity in the initial phases of the experiment. However, at the end of the incubation time (72 h), enzymatic synergism became apparent, as revealed by BG, CBH, BM, and BM assays. Specifically, the co-cultures displayed the following DS values: 6.4 (±3.9), 2.4 (±0.6), 4.8 (±2.6), 6.4 (5.7 ± 0.6), respectively (Supplementary Figures S2A–D). Clearly, the enhanced cell densities at later stages of incubation drove the strains to synergism also at the enzymatic level.

### Are Released Compounds at the Basis of the Synergism?

**FIGURE 4:**
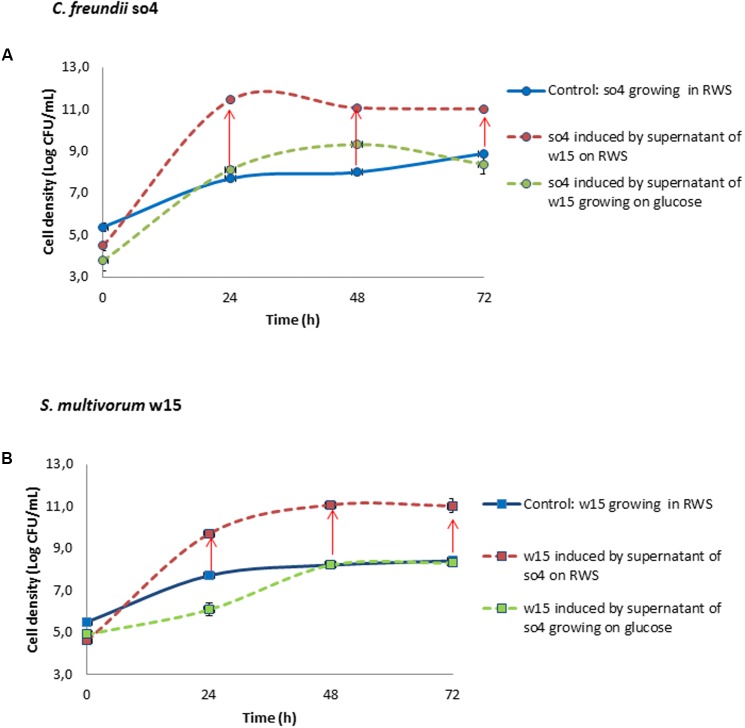
Induction experiment. Effect of supernatant from *C. freundii* so4 (circle), growing on raw wheat straw (RWS) or glucose, on the growth of *S. multivorum* w15 (square) and vice versa. In **(A)** *C. freundii* so4 is the recipient and *S. multivorum* w15 is the donor grown in monoculture on raw wheat straw (red) or glucose (green); supernatant from RWS-grown strain w15 produced a significant increase (*t*-test, *P* < 0.05) in the growth of *C. freundii* so4 (as compared to the control on RWS (blue)). In **(B)** *S. multivorum* w15 is the recipient and *C. freundii* so4 is the donor, grownin monoculture on RWS (red) or glucose (green); supernatant from RWS-grown strain so4 produced a significant increase (*t*-test, *P* < 0.05) in the growth of *S. multivorum* w15 (as compared to the control) on RWS (blue). Red arrow indicates synergistic growth. Standard deviation based on data from triplicate systems - (shown by vertical bars or within symbol dimensions).

To explore the mechanism involved in the synergism, we selected the *C. freundii* so4/*S. multivorum* w15 pair. Monocultures of each strain were treated with freshly harvested supernatants of their partner strain, in two different conditions. In the first case, both supernatant donor strains had been grown on raw wheat straw and in the second case on glucose. The supernatants originating from growth in the two different media affected partner strains to very different extents (**Figure 4**). Both partners of the pair revealed significant (*t*-test, *P* < 0.05) growth enhancements when treated with supernatants from the partner organism grown in raw wheat straw. However, this was not the case for the cultures grown in glucose. Below, we provide details of the growth and enzymatic potential parameters.

#### Growth

Upon addition of the supernatant of the counterpart strain grown in raw wheat straw, *C. freundii* so4 (growing on raw wheat straw) exhibited a biomass increase of 27.9 fold (±0.7) (**Figure 4A**) as compared to the respective control monoculture. *S. multivorum* w15 revealed a similar 24.9 (±2.7) fold biomass increment following induction (**Figure 4B**). In contrast, when supernatants were used from bacterial donors grown in glucose, *S. multivorum* w15 (growing on raw wheat straw) presented a longer log phase and a growth reduction of 45-fold (±4.1) at 24 h. However, at the end of the experiment (72 h), the strain reached the same biomass as the control (growing on raw wheat straw) (**Figure 4B**). *C. freundii* so4 growing on raw wheat straw and induced by the counterpart strain supernatant (growing in glucose) showed a slight (3.7 ± 0.1 fold) increase in biomass at 48 h. However, this strain had the same biomass as the control one at the end of the incubation (27 h) (**Figure 4A**).

#### Degradation Potential

Remarkably, the enzymatic activities in the cultures induced by supernatants of strains growing in glucose did not show significant differences from those in the control (uninduced) cultures in both cases (Supplementary Figure S3). In contrast, the enzymatic activities in the two cultures that had been treated with supernatants from raw wheat straw grown partner strains revealed an important difference, in both directions. The monocultures growing on raw wheat straw, at time zero, did not show any enzymatic activity in the four assays (β-glucosidases, cellobiohydrolases, β-mannosidases, and β-xylosidases) (data not shown). In contrast, upon treatment with supernatants from the RWS-grown partner, high enzymatic activities were found in all assays of the resulting supernatants as from the start of the culture, as compared to the untreated control. The impact on the activity was the same for both strains (*C. freundii* so4, *S. multivorum* w15) (Supplementary Figure S3).

The effect of the supernatants of donor *S. multivorum* w15 on *C. freundii* so4 was relatively constant, with somewhat increasing values along the culture time, compared with the control (Supplementary Figure S3). Thus, *S. multivorum* w15 presumably collaborates with *C. freundii* so4 by contributing diverse enzymatic activities. Conversely, *S. multivorum* w15 as a recipient of *C. freundii* so4 supernatant showed enhanced enzymatic activity only during the first 24 h of incubation. However, this did not impact the growth of w15, indicating that *C. freundii* so4 stimulates *S. multivorum* w15 temporarily by a mechanism different from enzymatic enhancement.

## Discussion

The interest in using co-cultures or consortia in the LCB bioprocess industry has increased recently. For instance, microbial consortia have been proposed as key agents in the degradation of wheat straw (Jiménez et al., 2013; Ghosh et al., 2016; Jia et al., 2016). The underlying assumption was that they provide a perfect mix of diverse lignocellulolytic enzymes required to degrade the recalcitrant compounds in wheat straw. In particular, metabolic cooperation between microorganisms and synergistic action of secreted enzymes may allow for an efficient degradation process (Taha et al., 2015; Jiménez et al., 2017). In this study, we aimed at characterizing to what extent cooperation between individual populations from the microbial consortia affects lignocellulose degradation, by characterizing co-cultures (in comparison to monocultures) of lignocellulose-degrading bacteria and fungi. The cultures were monitored through time, thus providing a dynamic view of both growth and enzyme activities. Our results clearly indicate that bacterial synergism does play a substantial role in subsets of organisms in such consortia and that the relationship between strains inhabiting the same system is dependent on the complexity of the carbon source.

### Metabolic Complementarity

Overall, a positive relationship was found between the abundance of particular degrading bacteria (in raw wheat straw derived consortia) and their capacity to grow on the substrate (**Figure 1** and Supplementary Figure S1). This finding corroborated the conclusion that the enrichment process used indeed allowed the selection of strains with high LCB degradative capacity. We further addressed the ability of selected lignocellulose degrading strains to establish a [positive] relationship with each other, as suggested in an earlier study (Cortes-Tolalpa et al., 2016). Synergistic interactions were indeed observed in five of 13 co-cultures, and metabolic complementarity of the component strains was invoked as the most likely mechanism involved. For instance, the most promising synergistic pair, composed of *C. freundii* so4 and *S. multivorum* w15 (biculture A) displayed superior growth in co-culture as compared to the respective monocultures, with synergistic activities of several hydrolytic enzymes (**Figure 2A**). *C. freundii* and *S. multivorum* differ widely in their metabolic properties. *C. freundii* is a member of the *Enterobacteriaceae*, a facultatively anaerobic family, with motility by flagella. It is able to grow on glycerol as well as citrate as sole carbon sources (Rosenberg et al., 2014a). *S. multivorum* belongs to the *Sphingobacteriaceae*. It is a strict aerobe, which does not possess flagellar motility. It is able to produce acid from a large variety of carbohydrates (including α-D-glucopyranoside and α-D-mannopyranoside) by oxidative processes. In fact, the organism is able to grow on *p*-hydroxy-butyrate as a single carbon source, but not on glycerol, like *C. freundii*. Moreover, *S. multivorum* is well known as a producer of extracellular enzymes, mainly xylosidases, proteases, and lipases (Rosenberg et al., 2014b). Both strains are capable of transforming cellobiose.

### Division of Labor

In our study, *S. multivorum* w15 probably contributes to cultures growing on wheat straw with efficient extracellular enzymes. In particular the release of different types of xylosidases seems to be a common feature among *S. multivorum* strains (Malfliet et al., 2013; Lian et al., 2016). Here, growing on raw wheat straw, *S. multivorum* w15 produced powerful cellobiohydrolases and β-xylosidases; such enzymes were not found with *C. freundii* so4 when grown under the same conditions (**Figure 2A**). We also found highly active β-xylosidases from *S. multivorum* strains w15 and so22, grown on wheat straw singly and in co-culture (**Figure 2** and Supplementary Figure S1). Moreover, it has been indicated that *S. multivorum* has lignin-degradation potential, which suggests the organism may also play a role in the degradation of the lignin present in wheat straw (Taylor et al., 2012). Such key metabolic activities allow *S. multivorum* to establish positive interactions with *C. freundii* so4. On the other hand, *C. freundii* so4 showed excellent growth on glucose, as opposed to *S. multivorum* w15. However, strain w15 did grow well in the glucose bicultures, which indicates that *C. freundii* so4 exerted a positive metabolic effect on its counterpart strain (**Figure 3**). We hypothesized that it probably provides redox power and contributes to the degradation of oligosaccharides to simpler sugars. This might be stimulated by its high motility, allowing it to explore the substrate. Furthermore, given the strict aerobic metabolism of *S. multivorum* w15, it is very likely that *C. freundii* so4 produces metabolic intermediates that *S. multivorum* w15 can consume, allowing it to reach higher cell densities in co-culture than in monoculture.

Furthermore, the observed growth stimulation of the *S. multivorum* w15 as well as the *C. freundii* so4 monocultures following treatment with the supernatant of the counterpart wheat-straw-grown strain further corroborates the contention that synergistic interactions take place when growing on wheat straw. We speculate that, in both cases, the recipient strain was capable of reaching increased cellular density after receiving, from the donor, a considerable number of secreted enzymes, next to (potentially) other compounds. With respect to the latter, signaling could be involved. This is corroborated by the fact that a quorum sensing system has been found in *C. freundii* (Rosenberg et al., 2014a; Wang and Zhou, 2015). Although we cannot precisely pinpoint the mechanisms that drive the interactions in our co-cultures, as well as the large increase of enzymatic activities observed in them (**Figures 1**, **2**), the supernatant-induced growth stimuli (**Figure 4**) provide clear evidence for synergistic interactions. Moreover, the metabolic differences between the two strains suggest that they divide ‘labor’ in the transformation of the heterogeneous wheat straw, allowing their co-cultures to build up an enhanced biomass. Importantly, the synergism was only observed with supernatants harvested from cells growing on raw wheat straw, but not with those from glucose-grown cells, indicating the relevance of the chemical complexity of the substrate (see below).

### Influence of the Carbon Source

The complexity of carbon sources can have a substantial influence on the metabolism of heterotrophic organisms (Deng and Wang, 2016). Klitgord and Segrè (2010), using flux balance analysis, found that different medium formulations (based on carbon, nitrogen, sulfur, and phosphorus) affect the interactions between microorganisms (Klitgord and Segrè, 2010). In our study, the more complex the substrate was, the more synergistic the relationship between *C. freundii* so4 and *S. multivorum* w15 became. Thus, the emergence of synergism in subsets of the original wheat-straw-grown microbial consortia can be linked to the inherent heterogeneity of the substrate, suggesting that the complexity of the carbon source can strongly modify the relationship between degrader strains. Specifically, we hypothesized that the level of synergism between bacteria involved in LCB degradation processes is related to the differential presence of bonds in substrates of different complexity. In the SLB, the three main components (cellulose, xylan, and lignin) were not tightly bound together in a matrix, such as was the case for the raw wheat straw. Thus, the finding that the collaborative bacterial pair showed synergism only at the end of the experiment is in line with this lower number of bonds (**Figure 3B**). Specifically, the presence of bonds between lignin and the complex carbohydrates cellulose and hemicellulose, or between them, may have been at the basis of the observed synergism. Such bonds determine to some extent the recalcitrance of the LCB (Du et al., 2014; Arnling Bååth et al., 2016). Notwithstanding our enhanced understanding of the bias of synergism and the link to recalcitrant bond numbers, further studies are necessary to understand this phenomenon in greater detail.

Overall, the data indicate that, when grown on raw wheat straw as the sole C and energy source, degradative strains first consume the labile parts of the substrate, after which they are in need to collaborate to access the remaining recalcitrant sources of carbon. We here posit that ‘multipolymer’ or ‘peeling’ synergism could be a model description of the mechanism involved in the synergism between *S. multivorum* w15 and *C. freundii* so4 on raw wheat straw. In this type of synergism, proposed by Selig et al. (2008) and Várnai et al. (2011), cellulose and hemicellulose are, at the same time, “peeled off” by enzymatic action, exposing new structures of the substrate to the hydrolytic enzymes that are or become available. For the complete hydrolysis of the raw wheat straw, different types of lignocellulolytic enzymes are probably required, in a temporally and spatially dynamic manner (Selig et al., 2008; Várnai et al., 2011).

### Final Remarks

Overall, this study reveals that, in LCB degradation processes, co-cultures of particular nature are superior to monocultures, as they allow division of labor in the metabolic processes that are required by the substrate. Clearly, microorganisms often lack some key metabolic pathways, which may be supplemented by others (Mikesková et al., 2012; Abreu and Taga, 2016; Ghosh et al., 2016). Thus, LCB degradation, in the end, may impose ‘group selection’ pressure on the process participants, in which ‘group’ is not defined by ‘kin’ but is rather determined by complementarity in a spatially- and temporally-explicit process. Our findings are consistent with recent data that show that co-cultures often present improved performance over corresponding monocultures. The mechanisms involved may include enhanced substrate utilization, overcoming of nutritional limitations, reduction of the levels of cheaters/scavengers and achieving superior overall activity, conversion and enzymatic action (Feng et al., 2011; Okeke and Lu, 2011; Zuroff et al., 2013; Liao et al., 2015; Valdez-Vazquez et al., 2015).

## Ethics Statement

This article does not contain any studies with human participants or animals performance by any of the other authors.

## Author Contributions

Conceptualization: LCT, JFS, and JDvE; formal analysis: LCT; funding acquisition: LCT and JDvE; investigation: LCT; methodology: LCT, JFS, and JDvE; supervision: JFS and JDvE; validation: JFS and JDvE; visualization: LCT, JFS, and JDvE; writing – original draft preparation: LCT; writing – review and editing: LCT, JFS, and JDvE.

## Conflict of Interest Statement

The authors declare that the research was conducted in the absence of any commercial or financial relationships that could be construed as a potential conflict of interest.

## References

[B1] Abreu N. A. Taga M. E. (2016). Decoding molecular interactions in microbial communities. *FEMS Microbiol. Rev.* 40 648–663. 10.1093/femsre/fuw019 27417261 PMC5007284

[B2] Aguirre-von-Wobeser E. Soberón-Chávez G. Eguiarte L. E. Ponce-Soto G. Y. Vázquez-Rosas-Landa M. Souza V. (2014). Two-role model of an interaction network of free-living γ-proteobacteria from an oligotrophic environment. *Environ. Microbiol.* 16 1366–1377. 10.1111/1462-2920.12305 24128119

[B3] Arnling Bååth J. Giummarella N. Klaubauf S. Lawoko M. Olsson L. (2016). A glucuronoyl esterase from *Acremonium alcalophilum* cleaves native lignin-carbohydrate ester bonds. *FEBS Lett.* 590 2611–2618. 10.1002/1873-3468.12290 27397104

[B4] Bhattacharya A. S. Bhattacharya A. Pletschke B. I. (2015). Synergism of fungal and bacterial cellulases and hemicellulases: a novel perspective for enhanced bio-ethanol production. *Biotechnol. Lett.* 37 1117–1129. 10.1007/s10529-015-1779-3 25656474

[B5] Brossi M. J. L. Jiménez D. J. Cortes-Tolalpa L. van Elsas J. D. (2015). Soil-derived microbial consortia enriched with different plant biomass reveal distinct players acting in lignocellulose degradation. *Microb. Ecol.* 71 616–627. 10.1007/s00248-015-0683-7 26487437 PMC4788684

[B6] Burkholder P. R. Pfister R. M. Leitz F. H. (1966). Production of a pyrrole antibiotic by a marine *bacterium1*. *Appl. Microbiol.* 14 649–653. 4380876 10.1128/am.14.4.649-653.1966 PMC546803

[B7] Chang J.-J. Chou C.-H. Ho C.-Y. Chen W.-E. Lay J.-J. Huang C.-C. (2008). Syntrophic co-culture of aerobic *Bacillus* and anaerobic *Clostridium* for bio-fuels and bio-hydrogen production. *Int. J. Hydrogen Energy* 33 5137–5146. 10.1016/j.ijhydene.2008.05.021

[B8] Cortes-Tolalpa L. Jiménez D. J. Brossi M. J. L. Salles J. F. van Elsas J. D. (2016). Different inocula produce distinctive microbial consortia with similar lignocellulose degradation capacity. *Appl. Microbiol. Biotechnol.* 100 7713–7725. 10.1007/s00253-016-7516-6 27170322 PMC4980425

[B9] Cragg S. M. Beckham G. T. Bruce N. C. Distel D. L. Dupree P. Etxabe A. G. (2015). Lignocellulose degradation mechanisms across the tree of life. *Curr. Opin. Chem. Biol.* 29 108–119. 10.1016/j.cbpa.2015.10.018 26583519 PMC7571853

[B10] Deng Y.-J. Wang S. Y. (2016). Synergistic growth in bacteria depends on substrate complexity. *J. Microbiol.* 54 23–30. 10.1007/s12275-016-5461-9 26727898 PMC4822414

[B11] Dolinšek J. Goldschmidt F. Johnson D. R. (2016). Synthetic microbial ecology and the dynamic interplay between microbial genotypes. *FEMS Microbiol. Rev.* 40 961–979. 10.1093/femsre/fuw024 28201744

[B12] Du X. Pérez-Boada M. Fernández C. Rencoret J. del Río J. C. Jiménez-Barbero J. (2014). Analysis of lignin–carbohydrate and lignin–lignin linkages after hydrolase treatment of xylan–lignin, glucomannan–lignin and glucan–lignin complexes from spruce wood. *Planta* 239 1079–1090. 10.1007/s00425-014-2037-y 24531838

[B13] Feng Y. Yu Y. Wang X. Qu Y. Li D. He W. (2011). Degradation of raw corn stover powder (RCSP) by an enriched microbial consortium and its community structure. *Bioresour. Technol.* 102 742–747. 10.1016/j.biortech.2010.08.074 20863696

[B14] Ghosh S. Chowdhury R. Bhattacharya P. (2016). Mixed consortia in bioprocesses: role of microbial interactions. *Appl. Microbiol. Biotechnol.* 100 4283–4295. 10.1007/s00253-016-7448-1 27037693

[B15] Guerriero G. Hausman J.-F. Strauss J. Ertan H. Siddiqui K. S. (2016). Lignocellulosic biomass: biosynthesis, degradation, and industrial utilization. *Eng. Life Sci.* 16 1–16. 10.1002/elsc.201400196

[B16] Jia X. Liu C. Song H. Ding M. Du J. Ma Q. (2016). Design, analysis and application of synthetic microbial consortia. *Synth. Syst. Biotechnol.* 1 109–117. 10.1016/j.synbio.2016.02.001 PMC5640696 29062933

[B17] Jiang L.-L. Zhou J.-J. Quan C.-S. Xiu Z.-L. (2017). Advances in industrial microbiome based on microbial consortium for biorefinery. *Bioresour. Bioprocess.* 4 11. 10.1186/s40643-017-0141-0 28251041 PMC5306255

[B18] Jiménez D. J. Dini-Andreote F. DeAngelis K. M. Singer S. W. Salles J. F. van Elsas J. D. (2017). Ecological insights into the dynamics of plant biomass-degrading microbial consortia. *Trends Microbiol.* 10.1016/j.tim.2017.05.012 [Epub ahead of print]. 28648267

[B19] Jiménez D. J. Korenblum E. van Elsas J. D. (2013). Novel multispecies microbial consortia involved in lignocellulose and 5-hydroxymethylfurfural bioconversion. *Appl. Microbiol. Biotechnol.* 98 2789–2803. 10.1007/s00253-013-5253-7 24113822

[B20] Klitgord N. Segrè D. (2010). Environments that induce synthetic microbial ecosystems. *PLOS Comput. Biol.* 6:e1001002. 10.1371/journal.pcbi.1001002 21124952 PMC2987903

[B21] Lian J. Choi J. Tan Y. S. Howe A. Wen Z. Jarboe L. R. (2016). Identification of soil microbes capable of utilizing cellobiosan. *PLOS ONE* 11:e0149336. 10.1371/journal.pone.0149336 26872347 PMC4752346

[B22] Liao X. Chen C. Zhang J. Dai Y. Zhang X. Xie S. (2015). Dimethylamine biodegradation by mixed culture enriched from drinking water biofilter. *Chemosphere* 119 935–940. 10.1016/j.chemosphere.2014.09.020 25280176

[B23] Malfliet S. Justé A. Crauwels S. Willems K. De Cooman L. Lievens B. (2013). Assessing the xylanolytic bacterial diversity during the malting process. *Food Microbiol.* 36 406–415. 10.1016/j.fm.2013.06.025 24010623

[B24] Mikesková H. Novotný C. Svobodová K. (2012). Interspecific interactions in mixed microbial cultures in a biodegradation perspective. *Appl. Microbiol. Biotechnol.* 95 861–870. 10.1007/s00253-012-4234-6 22733114

[B25] Minty J. J. Singer M. E. Scholz S. A. Bae C.-H. Ahn J.-H. Foster C. E. (2013). Design and characterization of synthetic fungal-bacterial consortia for direct production of isobutanol from cellulosic biomass. *Proc. Natl. Acad. Sci. U.S.A.* 110 14592–14597. 10.1073/pnas.1218447110 23959872 PMC3767521

[B26] Mitri S. Foster K. R. (2013). The genotypic view of social interactions in microbial communities. *Annu. Rev. Genet.* 47 247–273. 10.1146/annurev-genet-111212-133307 24016192

[B27] Okeke B. C. Lu J. (2011). Characterization of a defined cellulolytic and xylanolytic bacterial consortium for bioprocessing of cellulose and hemicelluloses. *Appl. Biochem. Biotechnol.* 163 869–881. 10.1007/s12010-010-9091-0 20859703

[B28] Pandhal J. Noirel J. (2014). Synthetic microbial ecosystems for biotechnology. *Biotechnol. Lett.* 36 1141–1151. 10.107/s10529-014-1480-y 24563311

[B29] Pérez-Gutiérrez R.-A. López-Ramírez V. Islas Á. Alcaraz L. D. Hernández-González I. Olivera B. C. L. (2013). Antagonism influences assembly of a *Bacillus* guild in a local community and is depicted as a food-chain network. *ISME J.* 7 487–497. 10.1038/ismej.2012.119 23096405 PMC3578566

[B30] Rosenberg E. DeLong E. F. Lory S. Stackebrandt E. Thompson F. L. (eds) (2014a). *The Prokaryotes: Gamma Proteobacteria*, 4th Edn Berlin: Springer.

[B31] Rosenberg E. DeLong E. F. Lory S. Stackebrandt E. Thompson F. L. (eds) (2014b). *The Prokaryotes: Other Major Lineages of Bacteria and the Archaea*, 4th Edn Berlin: Springer 10.1007/978-3-642-38954-2

[B32] Selig M. J. Knoshaug E. P. Adney W. S. Himmel M. E. Decker S. R. (2008). Synergistic enhancement of cellobiohydrolase performance on pretreated corn stover by addition of xylanase and esterase activities. *Bioresour. Technol.* 99 4997–5005. 10.1016/j.biortech.2007.09.064 18006303

[B33] Taha M. Shahsavari E. Al-Hothaly K. Mouradov A. Smith A. T. Ball A. S. (2015). Enhanced biological straw saccharification through coculturing of lignocellulose-degrading microorganisms. *Appl. Biochem. Biotechnol.* 175 3709–3728. 10.1007/s12010-015-1539-9 25724976

[B34] Taylor C. R. Hardiman E. M. Ahmad M. Sainsbury P. D. Norris P. R. Bugg T. D. H. (2012). Isolation of bacterial strains able to metabolize lignin from screening of environmental samples. *J. Appl. Microbiol.* 113 521–530. 10.1111/j.1365-2672.2012.05352.x 22642383

[B35] Trifonova R. Postma J. van Elsas J. D. (2009). Interactions of plant-beneficial bacteria with the ascomycete *Coniochaeta ligniaria*. *J. Appl. Microbiol.* 106 1859–1866. 10.1111/j.1365-2672.2009.04163.x 19298515

[B36] Väisänen T. Haapala A. Lappalainen R. Tomppo L. (2016). Utilization of agricultural and forest industry waste and residues in natural fiber-polymer composites: a review. *Waste Manag.* 54 62–73. 10.1016/j.wasman.2016.04.037 27184447

[B37] Valdez-Vazquez I. Pérez-Rangel M. Tapia A. Buitrón G. Molina C. Hernández G. (2015). Hydrogen and butanol production from native wheat straw by synthetic microbial consortia integrated by species of *Enterococcus* and *Clostridium*. *Fuel* 159 214–222. 10.1016/j.fuel.2015.06.052

[B38] Van Dyk J. S. Gama R. Morrison D. Swart S. Pletschke B. I. (2013). Food processing waste: problems, current management and prospects for utilisation of the lignocellulose component through enzyme synergistic degradation. *Renew. Sustain. Energy Rev.* 26 521–531. 10.1016/j.rser.2013.06.016

[B39] Van Dyk J. S. Pletschke B. I. (2012). A review of lignocellulose bioconversion using enzymatic hydrolysis and synergistic cooperation between enzymes — Factors affecting enzymes, conversion and synergy. *Biotechnol. Adv.* 30 1458–1480. 10.1016/j.biotechadv.2012.03.002 22445788

[B40] Várnai A. Huikko L. Pere J. Siika-aho M. Viikari L. (2011). Synergistic action of xylanase and mannanase improves the total hydrolysis of softwood. *Bioresour. Technol.* 102 9096–9104. 10.1016/j.biortech.2011.06.059 21757337

[B41] Wang Y. Zhou J. (2015). Draft genome sequence of *Citrobacter freundii* strain ST2, a γ-proteobacterium that produces N-acylhomoserine lactones. *Genomics Data* 6 234–236. 10.1016/j.gdata.2015.10.003 26697383 PMC4664774

[B42] Wang Z. Cao G. Zheng J. Fu D. Song J. Zhang J. (2015). Developing a mesophilic co-culture for direct conversion of cellulose to butanol in consolidated bioprocess. *Biotechnol. Biofuels* 8 84. 10.1186/s13068-015-0266-3 26089984 PMC4471926

[B43] Zuroff T. R. Barri Xiques S. Curtis W. R. (2013). Consortia-mediated bioprocessing of cellulose to ethanol with a symbiotic *Clostridium phytofermentans*/yeast co-culture. *Biotechnol. Biofuels* 6:59. 10.1186/1754-6834-6-59 23628342 PMC3653780

