# Supplementary material for: Bacterial Synergism in Lignocellulose Biomass Degradation – Complementary Roles of Degraders As Influenced by Complexity of the Carbon Source
Source: Front Microbiol. 2017 Oct 10;8:1628. doi: 10.3389/fmicb.2017.01628 (PMC5641323; doi:10.3389/fmicb.2017.01628)
Supplement: Supplementary file 1 [file Image_1.pdf]

Supplementary Material

Bacterial synergism in lignocellulose biomass degradation - complementary roles of degraders as influenced by complexity of the carbon source

Larisa Cortes-Tolalpa*, Joana Falcao Salles, Jan Dirk van Elsas.

***Correspondence author:** Larisa Cortes-Tolalpa
l.cortes.tolalpa@gmail.com.

Supplementary Figure 1

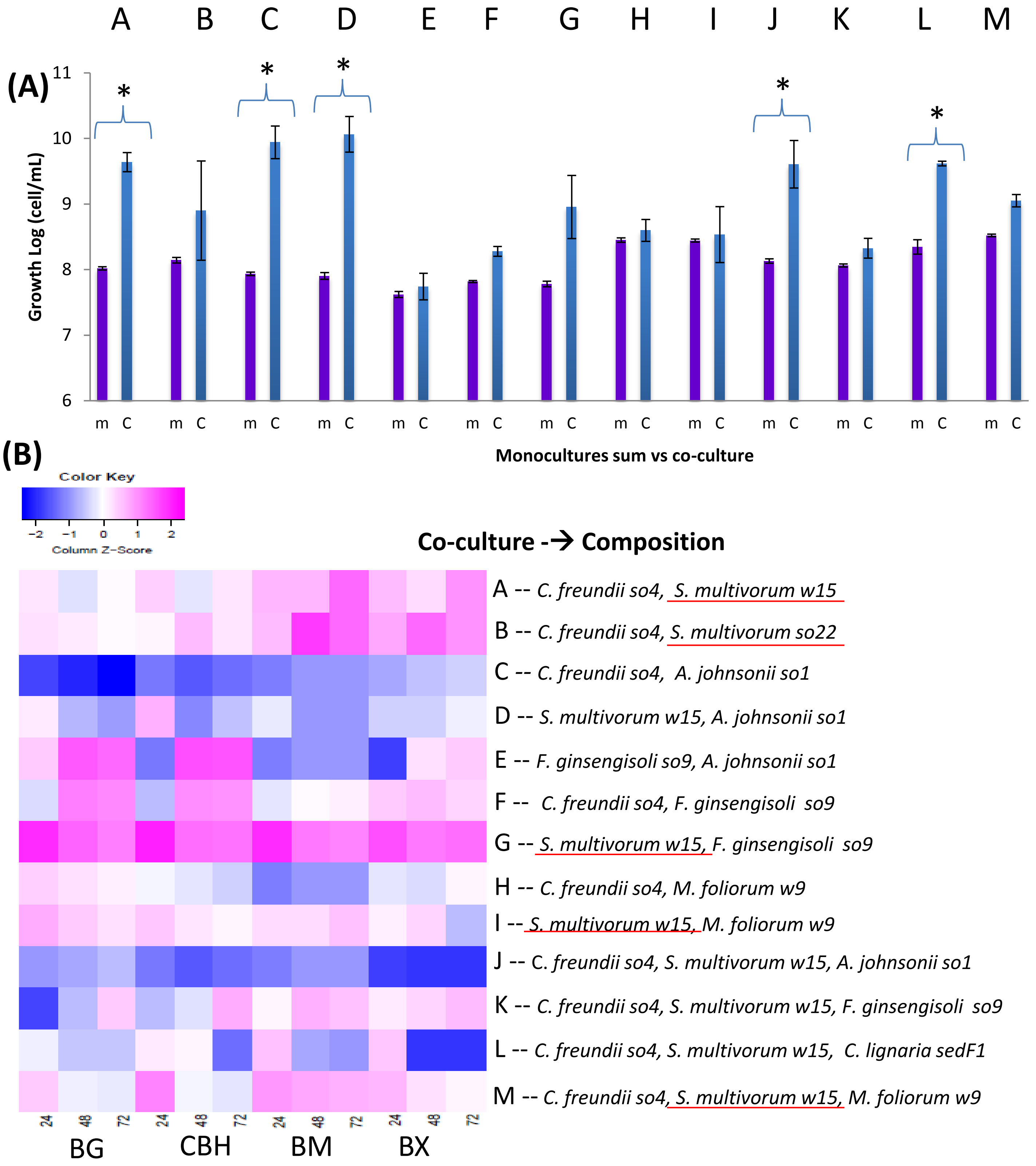

In (A) is shown the sum of growth from monocultures (purple) and co-cultures (blue), significant differences between the sum of monocultures and co-culture, t-test ($P<0.05$). Standard deviation correspond to triplicate. In (B) heatmap that displayed co-culture average (from triplicates) and normalized enzymatic activities BG: β-glucosidases, CBH: cellobiohydrolases, BM: β-mannosidases and BX: β-xylosidases, along the incubation time (24-72h), relative enzymatic activity reported in nmol MUB per h at 28°C, pH 6.8.

Supplementary Figure 2

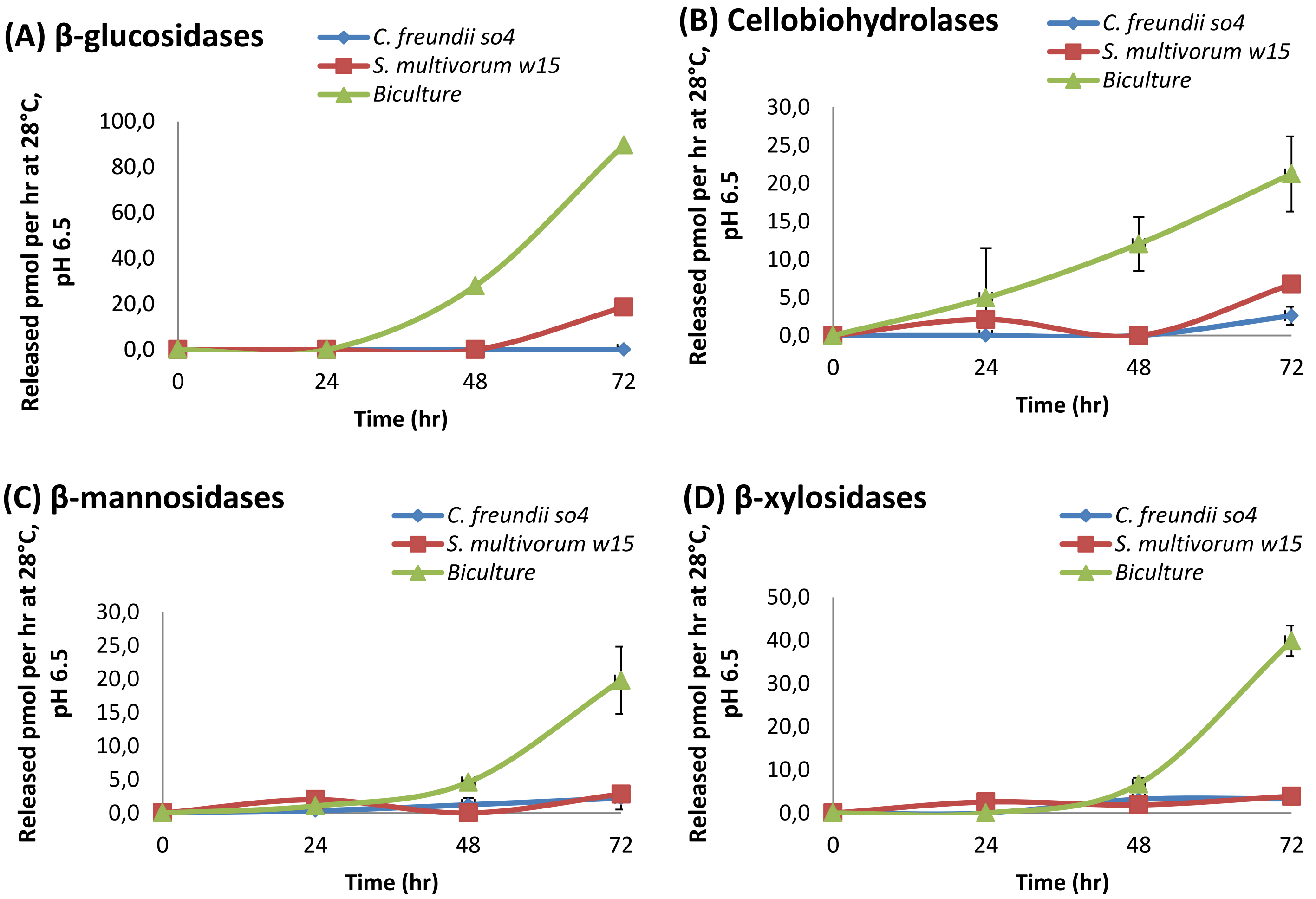

S2 Figure. Enzymatic activities from *S. multivorum* w15 and *C. freundii* so4 growing in synthetic recalcitrant biomass. (A) β-glucosidases, (B) cellobiohydrolases, (C) β-mannosidases, (D) β-xylosidases enzymatic activity from monocultures *S. multivorum* w15 (red) and *C. freundii* so4 (blue) and the co-culture (w15, so4) (green). Standard deviation correspond to triplicate systems.

Supplementary Figure 3

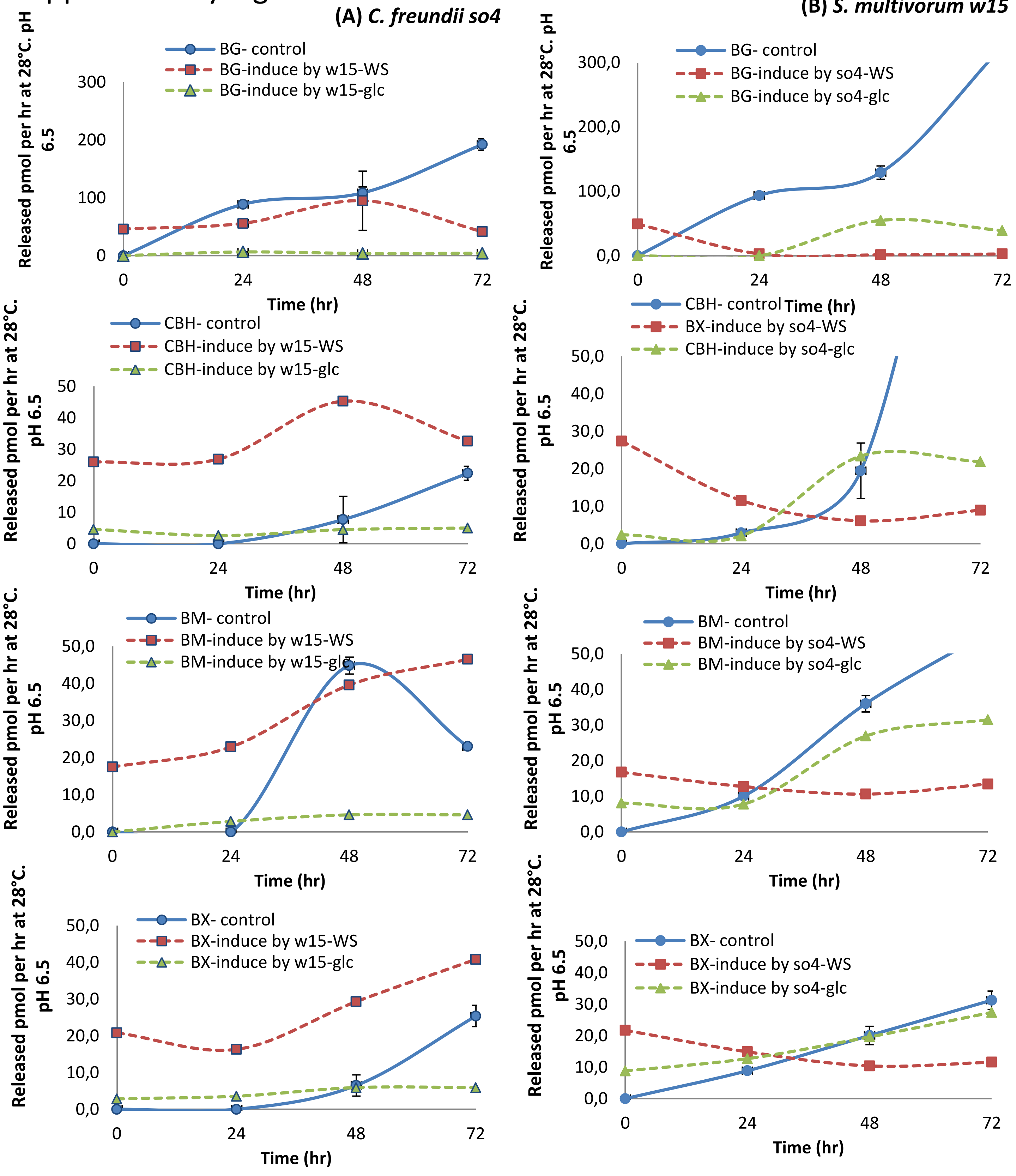

S3 Figure. Enzymatic activities from induction experiment. β-glucosidases, cellobiohydrolases, β-mannosidases and β-xylosidases activities (secretome) from (A) *C. freundii* so4 induced by supernatant from *S. multivorum* w15 and (B) *S. multivorum* w15 induce by supernatant of *C. freundii* so4. The donor strains was grown on glucose (green), grown on RWS (red). In blue the each strains grown on RWS as a control Standard deviation correspond to triplicate systems.
